# Ciliary body size in chronic angle-closure glaucoma

**DOI:** 10.1038/s41598-023-44085-8

**Published:** 2023-10-07

**Authors:** Jost B. Jonas, Rahul A. Jonas, Shefali B. Jonas, Songhomitra Panda-Jonas

**Affiliations:** 1 Department of Ophthalmology, Medical Faculty Mannheim, University Heidelberg, Kutzerufer 1, 68167 Mannheim, Germany; 2 Institute of Molecular and Clinical Ophthalmology Basel, Basel, Switzerland; 3 Singapore Eye Research Institute, Singapore, Singapore; 4 Department of Ophthalmology, University of Cologne, Cologne, Germany; 5 Hannover Medical School, Hannover, Germany; 6 Privatpraxis Prof Jonas Und Dr Panda-Jonas, Heidelberg, Germany

**Keywords:** Eye diseases, Optic nerve diseases

## Abstract

To examine the size of the ciliary body stroma (CBS) in dependence of the morphology of the anterior chamber angle in enucleated human eyes, we histomorphometrically examined human enucleated eyes. The study included 107 eyes (with a mean axial length of 25.1 ± 2.8 mm (range 21.0–36.0 mm). The anterior chamber angle was open in 68 eyes and it was closed and endothelialized in 39 eyes. The maximal CBS width (541 ± 210 µm versus 59 ± 179 µm; *P* < 0.001) and the minimal CBS width (214 ± 107 µm versus 17 ± 55 µm; *P* < 0.001) and maximal ciliary muscle height (593 ± 557 µm versus 293 ± 111 µm; *P* = 0.001) were significantly smaller in the angle-closure group than in the open-angle group. Maximal CBS width increased with presence of an open anterior chamber angle (beta: 0.82; B: 517; 95% CI 435, 599; *P* < 0.001) and longer axial length (beta: 0.17; B: 18.2; 95% CI 4.2, 32.2; P = 0.01). Minimal CBS width increased with the presence of an open anterior chamber angle (beta: 0.48; B: 131; 95% CI 80.4, 181; P < 0.001) and a larger maximal ciliary muscle height (beta: 0.33; B: 0.28; 95% CI 0.12, 0.44; P = 0.001). Maximal ciliary muscle height correlated with the maximal CBS height (beta: 0.35; B: 0.53; 95% CI 0.25, 0.81; *P* < 0.001). The findings suggest that the CBS size is markedly smaller in eyes with a chronically closed endothelialized anterior chamber angle than in eyes with open angles. The tightening of the angle in eyes with angle-closure may prevent the access of aqueous humor not only to the trabecular meshwork but also to the ciliary body and may reduce the uveoscleral or uveovortex outflow pathway.

## Introduction

The physiologic outflow of aqueous humor occurs through the trabecular meshwork into Schlemm’s canal and through the uveoscleral, or unconventional, outflow pathway^1–6^. The transtrabecular outflow requires an open anterior chamber angle so that the aqueous humor has free access to the trabecular meshwork. In eyes with a closure of the anterior chamber angle, the peripheral iris is adherent to the peripheral corneal endothelium. In the chronic stage of an anterior chamber angle closure, the anterior iris surface is often endothelialized by a growth of corneal endothelial cells from the cornea onto the iris surface^7,8^. In eyes with a closed anterior chamber angle, the intraocular pressure (IOP) is increased since the peripheral anterior synechia covers the trabecular meshwork, preventing the aqueous humor from getting access to Schlemm’s canal. In a similar manner, one may assume that also the uveoscleral outflow is reduced, since the access of the aqueous humor to the anterior ciliary body stroma (CBS) and indirectly, to the posterior part of the uveoscleral outflow is blocked by the anterior synechia. In the discussion of the pathogenesis of the IOP rise in eyes with secondary anterior angle-closure, a blockade of the uveoscleral outflow has only rarely been considered^1–8^. If the notion of a blockade of the uveoscleral outflow by a closure of the anterior chamber angle is valid, one may postulate that eyes with an open anterior chamber angle and with a full access of the aqueous humor to the CBS have a loosely arranged and sponge-like CBS. In contrast, eyes with a blockage of the aqueous humor pathway to the ciliary body may have a condensed or collapsed CBS. We therefore conducted this histological study to assess the dimensions of the CBS in eyes with open anterior chamber angles and eyes with a secondary angle-closure.

## Methods

Enucleated human eyes were examined in the histological study. They had been removed due to causes such as malignant uveal melanomas or painful secondary angle-closure glaucoma. The Medical Ethics Committee II of the Medical Faculty Mannheim of the Heidelberg University confirmed that study design was in agreement with the guidelines laid down in the World Medical Association Declaration of Helsinki. The Medical Ethics Committee II of the Medical Faculty Mannheim of the Heidelberg University approved the study and waived the necessity of an informed written consent, since the globes had been removed 25 to 60 years before the study was planned.

As also described in greater detail in previous reports, the eyes had been fixed at once after surgical enucleation^9,10^. They were kept in the fixation solution consisting of 4% formaldehyde and 1% glutaraldehyde for one week at room temperature. The globe diameters were determined in the anterior–posterior direction, horizontal direction and vertical direction. A central part running from the corneal center through the optic nerve head and about 8 mm thick was removed from of the eyes, dehydrated in alcohol and imbedded in paraffin. Slides about 5–8 µm thick and running through the central part of the cornea and pupil and through the optic nerve head were stained by hematoxylin–eosin or using the Periodic-Acid-Schiff (PAS) method.

**Figure 1:**
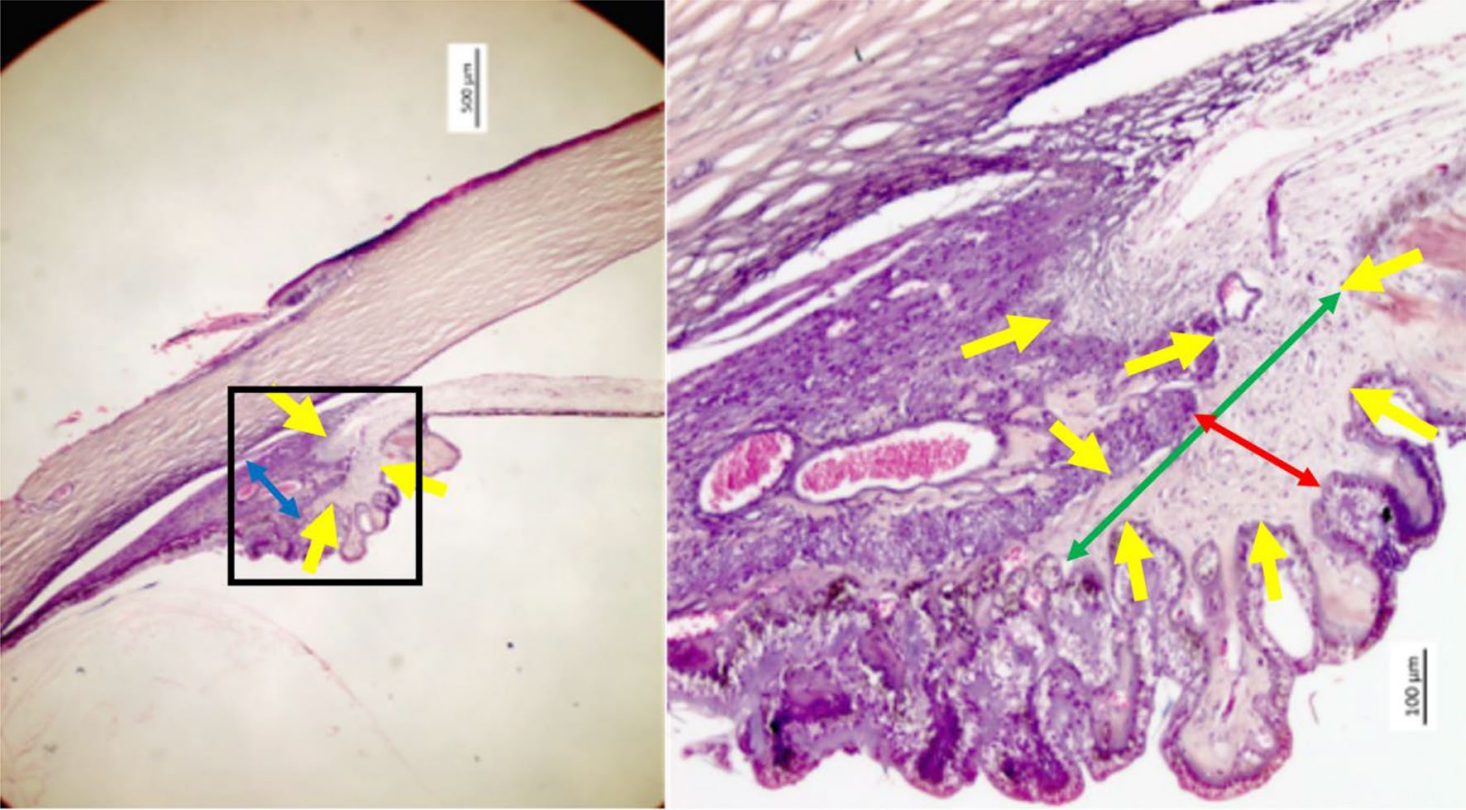
Histo-photograph showing the ciliary body of an enucleated human eye with open anterior chamber angle. Yellow arrows: Loosely arranged ciliary body stroma; blue double headed arrow: maximal thickness of the ciliary muscle; red double headed arrow: smallest width of the ciliary body stroma; green double headed arrow: largest width of the ciliary body stroma.

**Figure 2:**
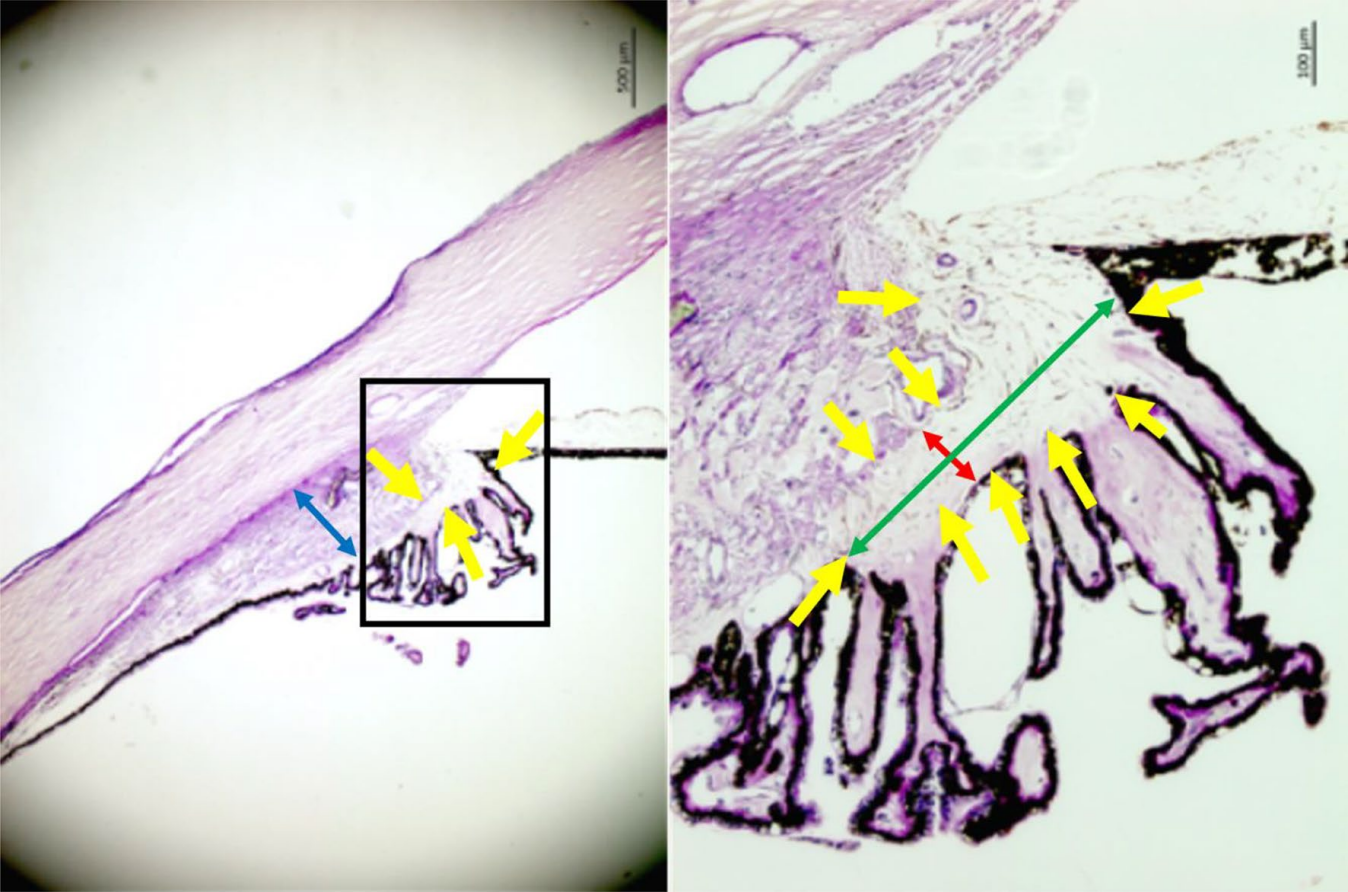
Histo-photograph showing the ciliary body of an enucleated human eye with open anterior chamber angle. Yellow arrows: Loosely arranged ciliary body stroma; blue double headed arrow: maximal thickness of the ciliary muscle; red double headed arrow: smallest width of the ciliary body stroma; green double headed arrow: largest width of the ciliary body stroma.

**Figure 3:**
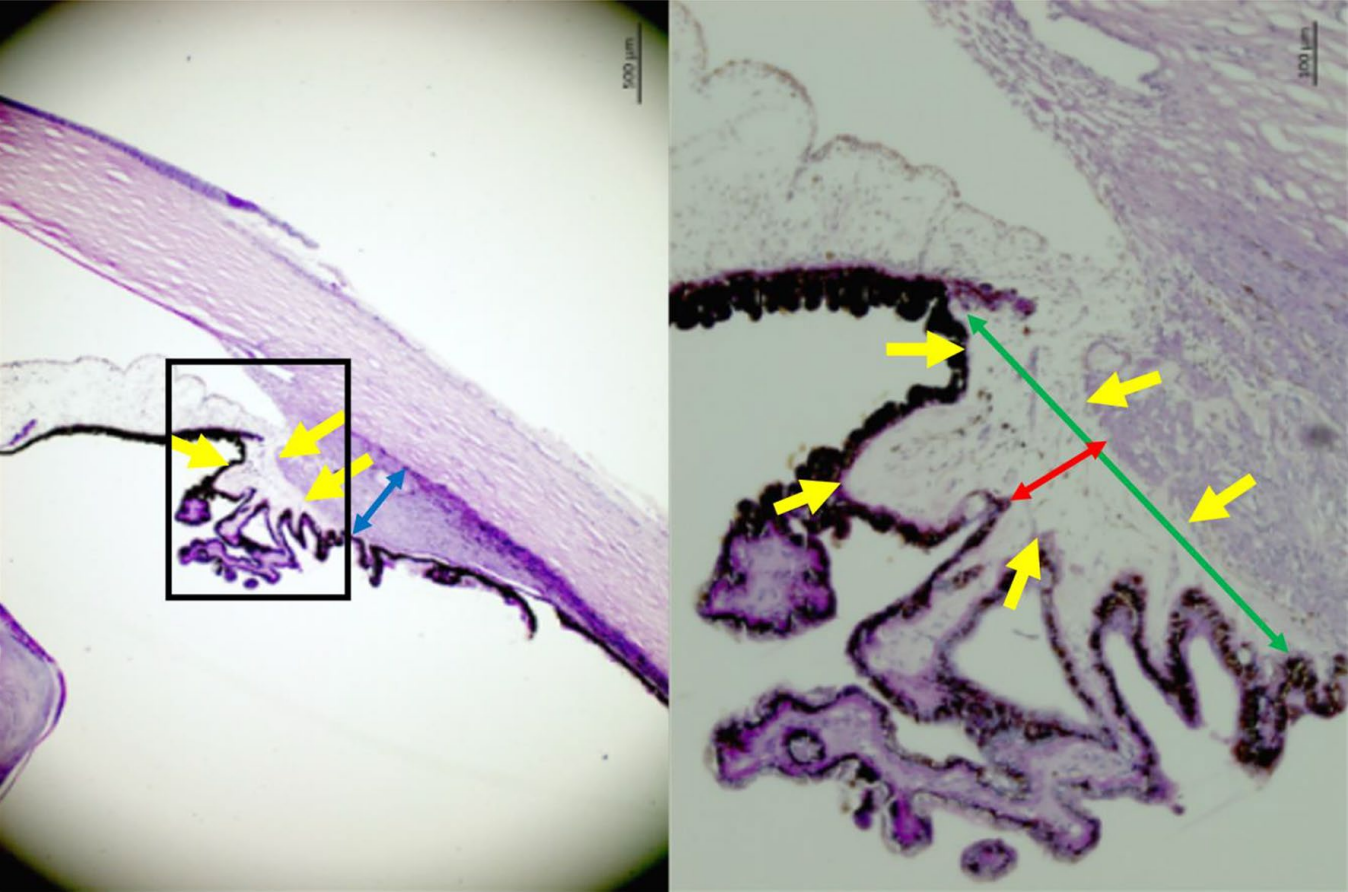
Histo-photograph showing the ciliary body of an enucleated human eye with open anterior chamber angle. Yellow arrows: Loosely arranged ciliary body stroma; blue double headed arrow: maximal thickness of the ciliary muscle; red double headed arrow: smallest width of the ciliary body stroma; green double headed arrow: largest width of the ciliary body stroma.

**Figure 4:**
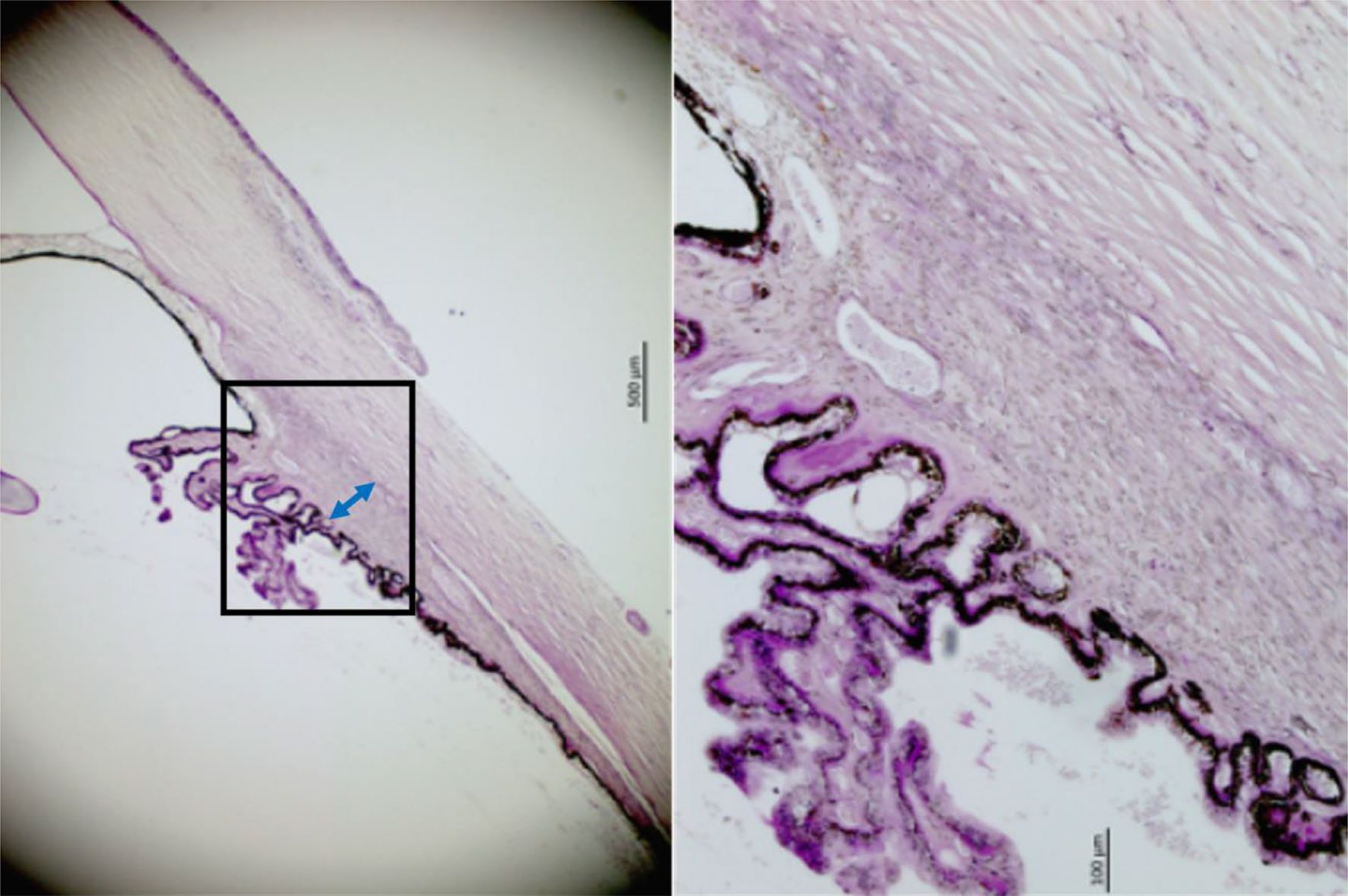
Histo-photograph showing the ciliary body of an enucleated human eye with an occluded anterior chamber angle. The ciliary body stroma is completely collapsed; maximal thickness of the ciliary muscle.

**Figure 5:**
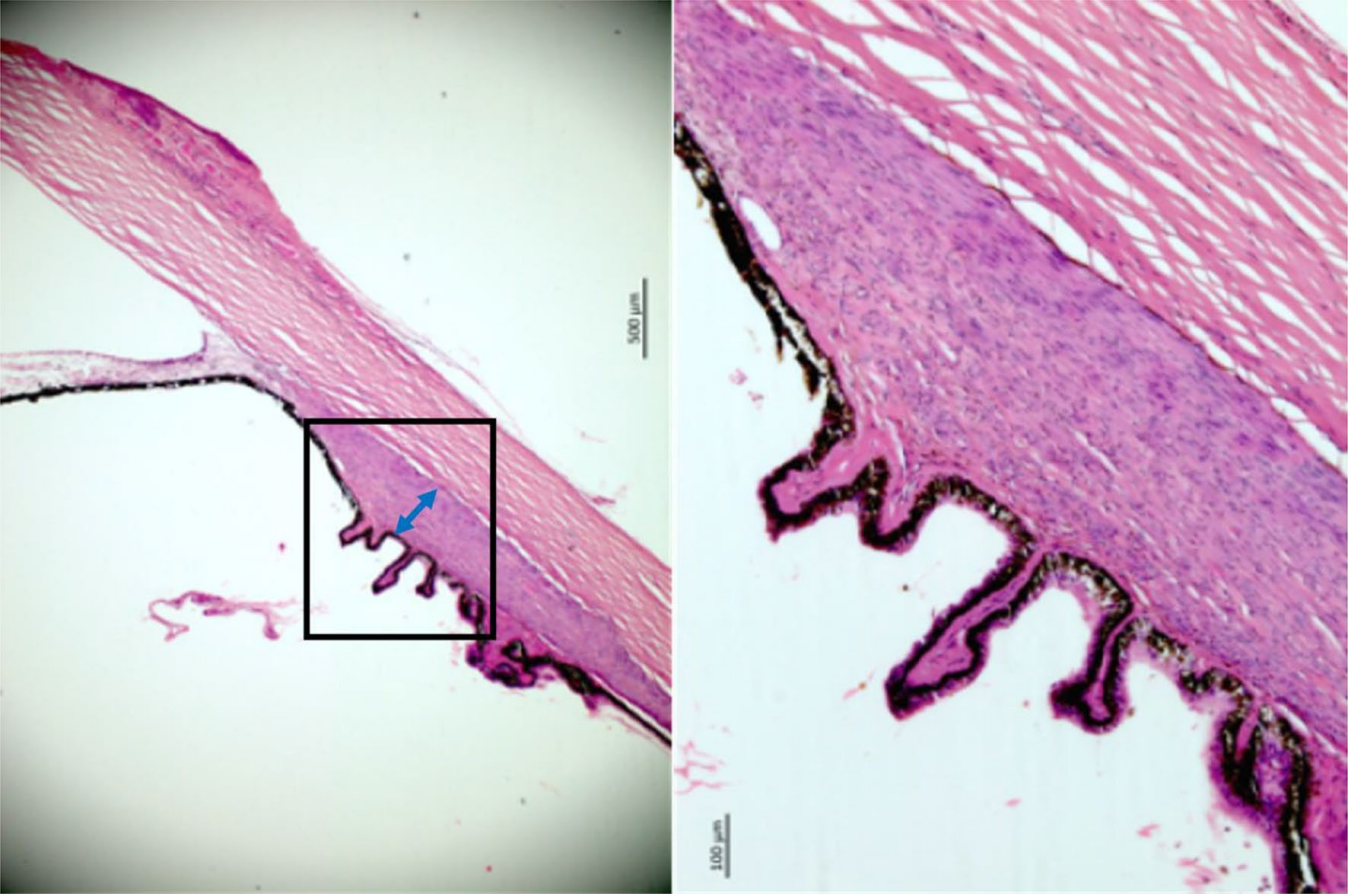
Histo-photograph showing the ciliary body of an enucleated human eye with an occluded anterior chamber angle. The ciliary body stroma is completely collapsed; maximal thickness of the ciliary muscle.

Using a millimeter scale built into the objective of the light microscope, we measured histomorphometrically the maximal width and minimal width of the CBS and the maximal height of the ciliary muscle (Figs. 1, 2, 3, 4, 5). As also described in detail previously, we also determined the length of the longitudinal part of the ciliary muscle, the distance between the end of the longitudinal part of the ciliary muscle and the ora serrata, the length of the pars plana region and pars plicata region of the ciliary body, and Bruch’s membrane thickness in the pars plana region. We noted the presence of cobble stones in the fundus periphery, macular Bruch’s membrane defects or and of scleral staphyloma^9^.

We differentiated between eyes with an open anterior chamber angle, in which the inner surface of the trabecular meshwork was exposed to the optically empty anterior chamber, and eyes with an occluded chamber angle, in which the peripheral iris was attached to the inner surface of the trabecular meshwork.

Using statistical software program (SPSS for Windows, version 27.0; IBM-SPSS, Chicago, Illinois, USA), we determined the mean values, standard deviations and 95% confidence intervals (CI) of the measured parameters. The parameters were compared with each other using the student t-test for paired samples, and they were compared between different groups, using the student t-test for unpaired samples. We additionally determined the standardized regression coefficient beta and the non-standardized regression coefficient B and its 95% confidence (CI) interval. The level of significance was 0.05 (two-sided).

## Results

The study included 107 eyes (61 men, 46 women) with a mean axial length of 25.1 ± 2.8 mm (range 21.0–36.0 mm) and a mean age of 61.2 ± 14.2 years (range 30–89 years). The mean age did not differ between the open-angle group and the angle-closure glaucoma group (59.7 ± 12.7 years versus 64.2 ± 17.5 years; *P* = 0.19) (Table 1). The anterior chamber angle was open in 68 eyes and it was closed in 39 eyes. In the eyes with a closed anterior chamber angle, the peripheral iris surface was endothelialized with a thin layer of cells on top of a PAS-positive membrane covering the iris surface, and these eyes showed characteristics of iris neovascularization. None of the eyes showed the morphology of a malignant glaucoma or ciliary block glaucoma. In none of the eyes, the ciliary body showed localized or segmental atrophy typical for a status after cyclocryocoagulation or cyclophotocoagulation.

**Table 1:** Histomorphometric measurements (mean ± standard deviation).

Parameter	Open anterior chamber angle	Closed anterior chamber angle	*P*-value
n	68	39	
Age (years)	59.7 ± 12.7	64.2 ± 17.5	0.19
Axial length (mm)	24.4 ± 2.4	26.4 ± 3.1	0.001
Ciliary body stroma, maximal width (µm)	541 ± 210	59 ± 179	< 0.001
Ciliary body stroma, minimal width (µm)	214 ± 107	17 ± 55	< 0.001
Ciliary muscle, maximal height (µm)	593 ± 557	293 ± 111	0.001

The maximal width (*P* < 0.001) and the minimal width (*P* < 0.001) of the CBS and the maximal height of the ciliary muscle (*P* = 0.001) were significantly smaller in the angle-closure group than in the open-angle group (Table 1).

In multivariable analysis, a longer maximal CBS width increased significantly with the presence of an open anterior chamber angle and longer axial length (Table 2). The following parameters were not significantly associated with maximal CBS width: age (*P* = 0.86), sex (*P* = 0.97), maximal height of the ciliary muscle (*P* = 0.62), length of the longitudinal part of the ciliary muscle (*P* = 0.29), distance between the end of the longitudinal part of the ciliary muscle and the ora serrata (*P* = 0.94), length of the pars plana region (*P* = 0.55) and pars plicata region (*P* = 0.27) of the ciliary body, total ciliary body length (*P* = 0.32), Bruch’s membrane thickness in the pars plana region (*P* = 0.64), and presence of cobble stones in the fundus periphery (*P* = 0.10), macular Bruch’s membrane defects (*P* = 0.08) and scleral staphyloma (*P* = 0.84) (Table 2).

**Table 2:** Associations (multivariable analysis) between the dimensions of the ciliary body stroma and other parameters.

Parameter	Standardized regression coefficient beta	Non-standardized regression coefficient B	95% Confidence interval of B	*P*-value
Ciliary body stroma, maximal width (mm)				
Anterior chamber angle open/occluded	0.82	517	435, 599	< 0.001
Axial length (mm)	0.17	18.2	4.2, 32.2	0.01
Parameters, which, when added separately to the model, were not significantly associated with maximal ciliary body stroma width				
Sex	0.003	2.00	− 117, 122	0.97
Age (years)	− 0.02	− 0.35	− 4.38, 3.68	0.86
Maximal ciliary body height	0.05	0.11	− 0.31, 0.52	0.62
Length of the longitudinal part of the ciliary muscle	0.14	0.04	− 0.04, 0.13	0.29
Distance between the end of the longitudinal part of the ciliary muscle and the ora serrata	0.01	0.01	− 0.14, 0.15	0.94
Length of pars plana region	0.08	0.02	− 0.05, 0.10	0.55
Length of pars plicata region	0.19	0.10	− 0.08, 0.28	0.27
Total ciliary body length	0.15	0.04	− 0.04, 0.11	0.32
Bruch’s membrane thickness in the pars plana region	0.04	9.91	− 32.9, 52.7	0.64
Presence of cobble stones in the fundus periphery	− 0.16	− 198	− 432, 36.2	0.10
Presence of macular Bruch’s membrane defects	− 0.15	− 161	− 342, 19.6	0.08
Presence of scleral staphyloma	− 0.02	− 30.9	− 330, 268	0.84
Ciliary body stroma, minimal width (mm)				
Anterior chamber angle open/occluded	0.48	131	80.4, 181	< 0.001
Maximal height of ciliary muscle (µm)	0.33	0.28	0.12, 0.44	0.001
Parameters, which, when added separately to the model, were not significantly associated with minimal ciliary body stroma width				
Sex	0.01	2.80	− 49.4, 55.0	0.92
Age (years)	0.05	0.42	− 1.35, 2.19	0.64
Axial length (mm)	0.09	4.31	− 1.97, 10.6	0.18
Length of the longitudinal part of the ciliary muscle	− 0.11	− 0.02	− 0.05, 0.02	0.36
Distance between the end of the longitudinal part of the ciliary muscle and the ora serrata	− 0.03	− 0.01	− 0.06, 0.05	0.79
Length of pars plana region	− 0.01	− 0.001	− 0.03, 0.03	0.97
Length of pars plicata region	− 0.26	− 0.06	− 0.12, 0.00	0.05
Total ciliary body length	− 0.12	− 0.01	− 0.04, 0.02	0.35
Bruch’s membrane thickness in the pars plana region	− 0.10	− 2.18	− 6.69, 2.32	0.33
Presence of cobble stones in the fundus periphery	− 0.05	− 24.7	− 93.2, 43.8	0.48
Presence of macular Bruch’s membrane defects	0.07	34.2	− 29.5, 98.0	0.29
Presence of scleral staphyloma	− 0.06	− 45.0	− 157, 66.8	0.43

The minimal CBS width was associated (multivariable analysis) with the presence of an open anterior chamber angle (*P* < 0.001) and a larger maximal height of the ciliary muscle (*P* = 0.001), while it was not significantly related with the parameters of sex (*P* = 0.92), age (*P* = 0.64), axial length (*P* = 0.18), length of the longitudinal part of the ciliary muscle (*P* = 0.36), distance between the end of the longitudinal part of the ciliary muscle and the ora serrata (*P* = 0.79), length of the pars plana region (*P* = 0.97) and pars plicata region (*P* = 0.05) of the ciliary body, total ciliary body length (*P* = 0.35), Bruch’s membrane thickness in the pars plana region (*P* = 0.33), and presence of cobble stones in the fundus periphery (*P* = 0.48), macular Bruch’s membrane defects (*P* = 0.29) or scleral staphyloma (*P* = 0.43) (Table 2).

In univariate analysis, the maximal height of the ciliary muscle was correlated with an open anterior chamber angle (beta: 0.31; B: 300; 95% CI 120, 479; *P* < 0.001) and with the maximal width of the CBS (beta: 0.35). In multivariable analysis, it was associated only with the maximal CBS width (beta: beta: 0.35; B: 0.53; 95% CI 0.25, 0.81; *P* < 0.001). After adjusting for the maximal CBS width, the maximal height of the ciliary muscle was not significantly associated with an open anterior chamber angle (*P* = 0.45), axial length (*P* = 0.51), age (*P* = 0.87), or any of the other parameters mentioned above.

## Discussion

In this histomorphometric study on enucleated human eyes, the CBS dimensions were significantly smaller in eyes with a chronically occluded anterior chamber angle as compared to eyes with an open chamber angle. The CBS size was independent of other histomorphometric parameters such as length of the ciliary body, pars plicata or pars plana, length of the longitudinal part of the ciliary muscle, and features of the posterior ocular segment.

Although numerous studies have examined the light microscopical and ultrastructural anatomy of the ciliary body including the ciliary muscle and the ciliary processes, the size of the CBS in general, and in dependence of the morphology of the anterior chamber angle in particular, has not extensively been explored yet, except for a study conducted by Ueno and Naumann in 1989^1–9,11–15^. Examining eyes with secondary glaucoma, mostly with an anterior chamber closure, Ueno and Naumann reported that the mean thickness of the ciliary muscle was smaller (P < 0.001) in the glaucomatous group than in a group of eyes with malignant uveal melanoma^15^. It is in agreement with the observations made in our study that the maximal height of the ciliary muscle was significantly higher in the eyes with open anterior chamber angle than in eyes with a closed angle (beta: 0.31; *P* < 0.001). In contrast to Ueno and Naumann, we did not find an association between ciliary body size and age, neither in the total study population nor stratified in subgroups with glaucoma and without glaucoma. In addition to Ueno’s study, we measured the size of the CBS, a parameter hitherto histomorphometrically unexplored. The pathway of the outflow of aqueous humor not passing through the trabecular meshwork has been described to occur in an “unconventional way”. The latter may include an uveoscleral pathway through the ciliary muscle and the supraciliary and suprachoroidal spaces across the sclera into the orbit, and an uveovortex pathway in which the aqueous humor is drained through the vortex veins, after passing the ciliary body and the supraciliary and suprachoroidal spaces^4^. The unconventional pathway was detected by Anders Bill who found radiolabeled albumin, when into the anterior chamber, in the uvea and sclera^16,17^. In addition, an additional lymphatic outflow of aqueous humor has been discussed^18–21^.

The results of the present study in which the CBS was significantly smaller in eyes with a chronically closed anterior chamber angle as compared to eyes with an open angle may suggest that the angle closure reduced the unconventional outflow of aqueous humor, in addition to the blockade of the transtrabecular outflow. It might have led to a “drying-up” of the CBS, due to a lack of inflow of aqueous humor into the CBS. This notion fits with the results of studies conducted by Sherman and colleagues who injected fluorescein into the anterior chamber of monkey eyes and observed a filling of the transtrabecular outflow pathway within five minutes after the fluorescein instillation^22^. In addition, they found that the fluorescein quickly moved into the iris stroma and CBS, and after penetration into the blood vessels in these regions, fluorescein could be found posteriorly in the equatorial choroidal and vortex veins. These observations also fit with the anatomy of the anterior surface of the iris and of the CBS, both of which are not covered by a water-impermeable membrane but are a loosely arranged tissue open to the anterior chamber compartment. The notion also agrees with the observations made by Bill who detected radioactively labelled albumin in the uvea after injection into the anterior chamber^16,17^. The finding suggests that a chronic occlusion of the anterior chamber angle may lead to reduction of the unconventional outflow pathway of aqueous humor, namely the uveoscleral or uveovortex outflow pathway, by blocking the access of the aqueous humor to the CBS.

The reason for the significantly longer axial length in the angle-closure group as compared to the open-angle group was that the angle closure was secondary to reasons such as iris neovascularization and as such independent of axial length (Table 1). In addition, the study participants were not recruited in a population-based manner but the eyes were taken from the ophthalmological histo-pathological archives.

Our study has limitations, in particular that serial sections of the eyes were not available; that the eyes included into the study were affected by various disorders as reasons for their enucleation, in particular malignant choroidal melanomas in the subgroup of eyes with an open-anterior chamber angle; that the findings cannot directly be transferred to eyes with an acute angle closure; that histological artefacts due to the processing of the histologic sections have to be taken into account; and that the patients included in our investigations were of European descent, so that the findings may not directly transferred onto eyes of patients of other ethnicities. In addition, the presumably high IOP in the angle-closure glaucoma group during in the months to years before the enucleation was carried out might have induced atrophy of various ocular tissues like the iris and also in CBS. A high IOP during the enucleation might have led to a more marked blood outflow in the eyes with angle-closure than the eyes with malignant melanoma. Besides the angle-closure itself, a high IOP might have also contributed to the atrophy of the CBS.

In conclusion, the CBS appears to be condensed in eyes with a chronically closed anterior chamber angle suggesting that the anterior irido-corneal synechia reduced or blocked the access of aqueous humor not only to the trabecular meshwork but also to the ciliary body and may have reduced the unconventional outflow pathway.

## Data Availability

The datasets used and/or analyzed during the current study available from the corresponding author on reasonable request.
